# Adamantanes for the treatment of neurodegenerative diseases in the presence of SARS-CoV-2

**DOI:** 10.3389/fnins.2023.1128157

**Published:** 2023-03-03

**Authors:** Roger F. Butterworth

**Affiliations:** Department of Medicine, University of Montreal, Montreal, QC, Canada

**Keywords:** neurodegenerative diseases, Parkinson’s disease, Alzheimer’s disease, Multiple sclerosis, traumatic brain injury, amantadine, memantine

## Abstract

Advent of the acute respiratory coronavirus SARS-CoV-2 has resulted in the search for novel antiviral agents and in the repurposing of existing agents with demonstrated efficacy against other known coronaviruses in the search for an agent with antiviral activity for use during the COVID-19 pandemic. Adamantanes including amantadine, rimantadine, and memantine have well-established benefit in the treatment of neurodegenerative diseases including Parkinson’s disease (PD), Alzheimer’s disease (AD) and fatigue related to Multiple sclerosis (MS) all of which are known comorbidities related to COVID-19 Moreover, results of basic pharmacological studies both *in vitro* and *in vivo* reveal that amantadine has the potential to inhibit SARS-CoV-2 *via* down-regulation of host-cell proteases resulting in impaired viral genome release into the host cell and *via* amantadine’s property as an NMDA receptor antagonist resulting in the prevention of the acute lung injury and respiratory distress that is characteristic of COVID-19. Cases suggestive of COVID-19 prophylaxis have been reported in patients with PD or MS or severe cognitive impairment treated in all cases for several months with an adamantane [amantadine or memantine] who were subsequently infected with SARS-CoV-2 confirmed by RT-PCR, and, in all cases, no signs of infectious disease were encountered. Amantadine is effective for the treatment of fatigue in MS and for the neurological complications of Traumatic Brain Injury (TBI).

## Introduction

Neurodegenerative diseases including Parkinson’s disease [PD], Alzheimer’s disease [AD], and Multiple sclerosis [MS] are increasingly considered to represent comorbidities in patients infected with SARS-CoV-2, the coronavirus responsible for the 2019 pandemic currently known as COVID-19. The presence of these disorders has the potential to impact negatively on the severity of symptoms of the infection as well as the efficacy of treatment strategies and on patient survival.

Members of the adamantane family of agents have established beneficial effects on neurodegenerative diseases that include amantadine [for PD and for the treatment of fatigue in MS], memantine [for AD] and amantadine for the treatment of the decreased levels of consciousness and cognitive/behavioral sequelae of traumatic brain injury [TBI]. Evidence supports the notion that SARS-CoV-2-infection of a patient with AD results in worsening of both conditions. On the other hand, treatment with amantadine has the potential to benefit both situations *via* distinct neurophysiologic and antiviral mechanisms. The present article reviews these issues in an evidence-based manner from basic mechanisms to results of systematic reviews and meta-analyses in support of therapeutic efficacy in these neurodegenerative diseases during the COVID-19 pandemic.

## Parkinson’s disease

Parkinson’s disease [PD] is an age-related neurodegenerative disease characterized by the progressive selective deterioration and ultimate death of dopaminergic neurons situated in substantia nigra of the basal ganglia. PD shares several common features with COVID-19 including age-dependency and co-morbidities that include cardiovascular disorders, obesity, and diabetes with the capacity to the impact of COVID-10 on Strategies implicated in PD patient care and, conversely on the effects of PD on immune status resulting in possible increases in severity of COVID-19 (Prasad et al., 2020). Other common features of COVID-19 such as fever, stress, and anxiety may have deleterious effects on tremor, gait, and dyskinesias in PD in addition to compromise of the efficacy of L-Dopa (Butterworth, 2020a). Moreover, enhancement of antibody responses to coronaviruses have been described in cerebrospinal fluid [CSF] samples of PD patients and substantia nigra is a brain structure that is susceptible to viral infections including the MHV-A59 coronavirus (Takahashi et al., 1995).

**FIGURE 1:**
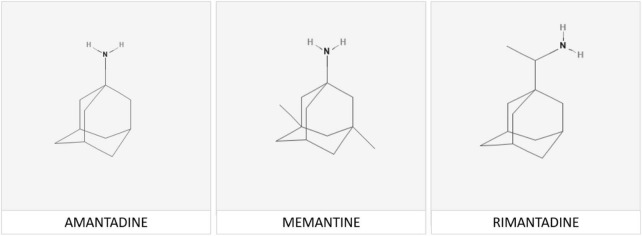
Memantine [adamantan-1-amine] is a member of the adamantane family of agents. The adamantane molecule is composed of three condensed cyclohexane ring structures fused together in an armchair configuration with a functional group substituted at one of the four methyne positions that determines the specificity of each individual compound. Chemical structures of memantine and its analogs rimantadine and amantadine are shown above.

The functional pathophysiologic links between PD, viral infection and adamantanes became evident following the publication of the serendipitous observation in a 68 year-old female patient with moderate-severe PD who, upon taking the adamantane compound amantadine for the treatment of influenza, noted a marked remission of her rigidity and tremor both of which reappeared upon cessation of amantadine. The molecular structures of amantadine and related adamantanes known to be effective for the treatment of neurodegenerative disease are shown in Figure 1.

Beneficial effects were confirmed in a subsequent clinical trial in 163 PD patients (Schwab et al., 1969) and is currently widely employed for the motor symptoms characteristic of PD and particularly for the treatment of L-Dopa-induced dyskinesias as demonstrated by meta-analysis of the results of several randomized clinical trials (Kong et al., 2017).

Enhancement of antibody responses to a range of coronaviruses have been reported in CSF samples from patients with PD and other evidence suggests that Parkinsonism is a common feature a range of viral encephalitides with associated regional neuropathology reminiscent of PD. For example, substantia nigral damage has been reported in association with the H1-N1 influenza virus and MHV-A59 coronaviral infection shows selective affinity for basal ganglia structures with accompanying postural and locomotor deficits resulting from neuronal cell death and gliosis in substantia nigra (Fishman et al., 1985) and a first case of meningitis/encephalitis associated with SARS-CoV-2 the virus responsible for the COVID-19 pandemic was reported in 2020 (Moriguchi et al., 2020).

**FIGURE 2:**
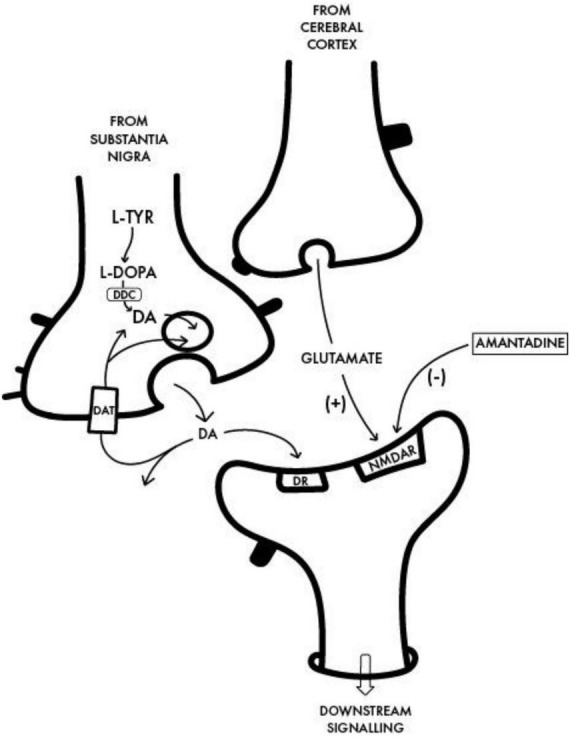
Interface between a dopaminergic nigrostriatal nerve terminal in which DA is synthesized from L-Tyrosine [L-TYR] *via* L-DOPA to dopamine with a glutamatergic terminal of the cortico-striatal tract and the postsynaptic neuron. The benefit of amantadine for the treatment of the motor disturbances in PD is attributed to its non-competitive antagonist action on the post-synaptic NMDA receptor [NMDAR] resulting in the restoration of the balance between nigrostriatal and corticostriatal inputs in favor of increased net production of DA. DDC, Dopa decarboxylase; DAT, dopamine transporter; DR, post-synaptic DA receptor.

Investigations into the potential beneficial effects of adamantanes against coronaviruses including SARS-CoV-2 continue at pace resulting in the discovery of novel mechanisms responsible for their neurotropic and neuroinvasive properties as well as those implicated in the protective effects of adamantanes (Butterworth, 2020b,^2021^). Other examples include studies of the human respiratory coronavirus HCoV-OC43, a strain known to activate neuroinflammatory and neurodegenerative processes in human neural cell populations cells leading to motor dysfunction and paralytic disease in virus-infected mice (Brison et al., 2014). Treatment with the adamantane analog memantine [structure shown in Figure 1) resulted in the reduction of viral replication rates together with improvements in survival times in a dose-dependent manner. Both amantadine and memantine are potent non-competitive antagonists of the N-Methyl-D-Aspartate [NMDA] subclass of receptor for glutamate, the principal excitatory amino acid neurotransmitter of mammalian brain (Figure 2). Over-activation of NMDA receptors has the potential to cause release of excess Ca++ and neuronal cell death. Similar mechanisms have been proposed to explain the pathogenesis of neuronal cell death in PD.

There is preliminary evidence from a series of published case reports that is suggestive of a protective effect of amantadine against infection by SARS-CoV-2. The study involved five PD patients all taking amantadine and L-Dopa for several weeks for treatment of motor symptoms of PD who tested positive for the virus by RT-PCR. None of the five patients went on to manifest symptoms of viral infection and improvements in motor function were maintained in all cases (Rejdak and Grieb, 2020). Confirmation of these interesting findings is now required under randomized controlled clinical trial conditions.

## Alzheimer’s disease

Alzheimer’s disease [AD] is a neurodegenerative disease characterized by acquired progressive memory loss with prevalence estimated of 25–30% in the population of Europe aged between the ages of 80 and 85 years in 1990. From a neurochemical pathologic standpoint, AD is characterized by significant neuronal cell loss from the nucleus basalis of Meynert resulting in a central cholinergic deficit. These conclusions led to intensive supplementary investigations and clinical trials of novel agents with the potential to restore the cholinergic deficit. One such group of compounds, the cholinesterase inhibitors having the appropriate structure/activity profile were initially shown to be beneficial from a symptomatic cognitive standpoint but were unfortunately shown to provide little by way of evidence in support of their use for the prevention of the neuronal cell damage and death characteristic of AD. On the other hand, results of a systematic review with meta-analysis of the results of 30 RCTs involving 7,567 patients demonstrated that the adamantane analog, memantine (Figure 1) was effective for the improvement of cognitive function in patients with AD compared to placebo, a finding that was highly significant either with or without the addition of cholinesterase inhibitors (Kishi et al., 2017). The case for the use of memantine progressed to gain FDA approval for the treatment of moderate-to-severe AD in the same year.

In recent years, several mechanisms have been proposed to account for the efficacy of memantine for the treatment of AD. Like amantadine, memantine is a potent NMDA receptor antagonist with the potential to inhibit the excess release of Ca^++^ following receptor activation as described above. Alternatively [or additionally], memantine is an established anti-inflammatory agent acting by attenuation of microglial activation known to be of key importance in the pathogenesis of neuronal cell death in AD (Wu et al., 2009).

Memantine, in common with other adamantanes, is effective against numerous viral infections including the human coronavirus HCoV-OC43 and an ongoing thesis proposes that the Herpes Simplex Virus Type 1 [HSV-1] rather than p-tau is responsible for the inter-neuronal trans-synaptic pathological cascade proposed for the inter-cerebral propagation of AD (Ball et al., 2013).

Evidence of functional links between AD and COVID-19 continues to accumulate. In common with other neurodegenerative diseases, AD is considered a co-morbidity for COVID-19 and the presence of one of the conditions frequently results in worsening of the other (Xia et al., 2021). Each condition results in neurocognitive impairment and neurodegeneration that is linked to the accumulation of amyloid precursor protein [APP] as well as to NMDA receptor activation and, since they share proinflammatory signaling cascades, neuronal cell dysfunction and loss has been attributed to microglial-mediated responses in both conditions (Butterworth, 2022). In relation to these mechanistic considerations, it is interesting to note that amyloid-beta oligomers are known to transit into the plasma membrane leading to the formation of pores that favor the passage of Ca^++^ following activation of NMDA receptors. It is interesting in this regard that both AD and COVID-19 appear to derive therapeutic benefit from treatment with the potent NMDA receptor antagonist memantine. Memantine exerts dose-dependent antiviral and neuroprotective against the human respiratory neuroinvasive coronavirus HCoV-OC43, a relative of SARS-CoV-2 where the beneficial effects were attributed to the reduction of microglial activation. Moreover, in relation to inflammatory responses, AD-related neuroinflammation coupled with that resulting from SARS-CoV-2 infection has the potential to result in a “cytokine storm” leading to extremely poor clinical outcomes in both situations.

**FIGURE 3:**
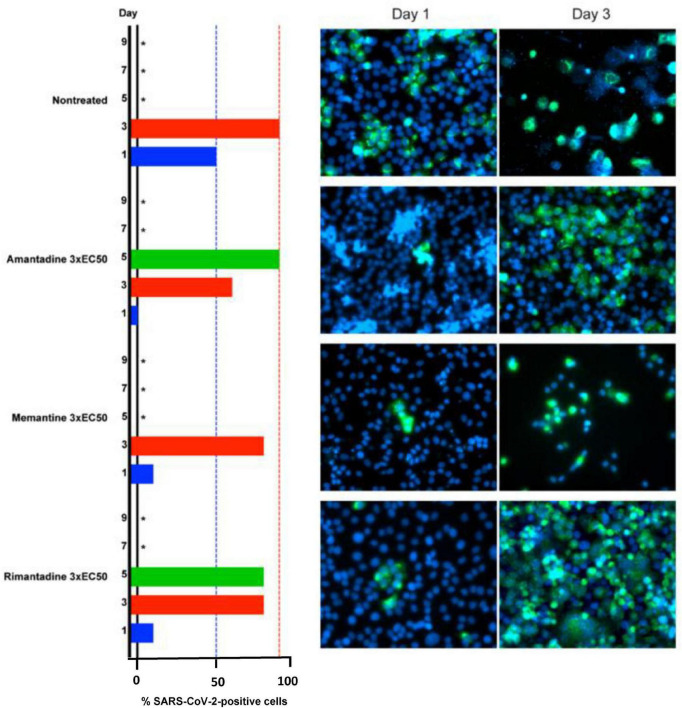
Barrier to SARS-CoV-2 escape by memantine, amantadine and rimantadine in their roles as ion channel inhibitors in Vero E6 cells at concentrations of 3 x EC50 on days 1,3,5,7,9 post-infection. Infected cells identified by immunostaining for SARS-CoV-2 spike protein (green) relative to counterstaining of cell nuclei with Hoechst dye (blue). *Not recorded/cell death.

Studies of the effects of adamantanes on the SARS-CoV-2 virus *per se* continue apace. In one such investigation, the antiviral actions of memantine, amantadine and rimantadine were compared in Vero E6 cells; results are shown in Figure 3. While all three analogs were effective, rimantadine was the most potent showing the highest selectivity index (Zhou et al., 2021). Mechanisms responsible for the antiviral properties of these agents include blocking of the viroporin channel of the E protein of SARS-CoV-2 leading to prevention of release of the viral nucleus into the host cell cytoplasm (Singh Tomar and Arkin, 2020) as well as down-regulation in expression of host cell proteases such as Cathexin L (Smieszek et al., 2020) and targeting ion channels encoded by the virus (Toft-Bertelsen et al., 2021).

## Multiple sclerosis

Central fatigue in Multiple sclerosis [MS] has a significant negative impact on disability scores and health-related quality of life [HRQOL] that occurs in patients with MS with increased severity and frequency in those with primary or secondary progressive disease compared to those with a relapsing-remitting presentation. Modern neuroimaging and spectroscopic techniques continue to support the thesis that predominantly centrally mediated mechanisms underpin the pathogenesis of fatigue in MS. Both the burden of Magnetic Resonance Imaging [MRI] lesions and abnormalities of motor-evoked potentials [MEPs] correlate in an independent manner with fatigue severity in MS patients consistent with its central origin (Colombo et al., 2000). In addition, region-selective cerebral metabolic dysfunction was confirmed using 18-fluorodeoxyglucose Positron Emission Tomography [PET] (Roelcke et al., 1997) and functional 1-H-Magnetic Resonance Spectroscopy [MRS] (DeLuca et al., 2008). Brain regions implicated included basal ganglia and frontal cortex giving credence to the notion that functional modifications of the striatal-thalamic-frontal cortical network play a key role in MS-related fatigue (Genova et al., 2013). Possible central mediators proposed include the neurotransmitter dopamine and the pro-inflammatory cytokine tumor necrosis factor alpha [TNFα].

Medications currently employed for the treatment of fatigue in MS. Amantadine is one such agent where results of 11 RCTs some comparing its efficacy to placebo, others comparing amantadine to that of other agents that included modafinil, pemoline, L-carnitine, ondansetron or methylphenidate were published resulting in six systematic reviews with two associated meta-analyses. The majority of cases confirmed that amantadine provided significant degrees of relief from fatigue in patients with either chronic persistent MS or in relapsing-remitting forms of the disorder. The consistency of these results contributed to the recommendations from the clinical practice guidelines published by The Royal College of Physicians: Multiple Sclerosis [NICE, UK) and by The German Society of Multiple Sclerosis recommending that amantadine be employed for the effective treatment of fatigue in MS (Pilling and Butterworth, 2021).

## Traumatic brain injury

**FIGURE 4:**
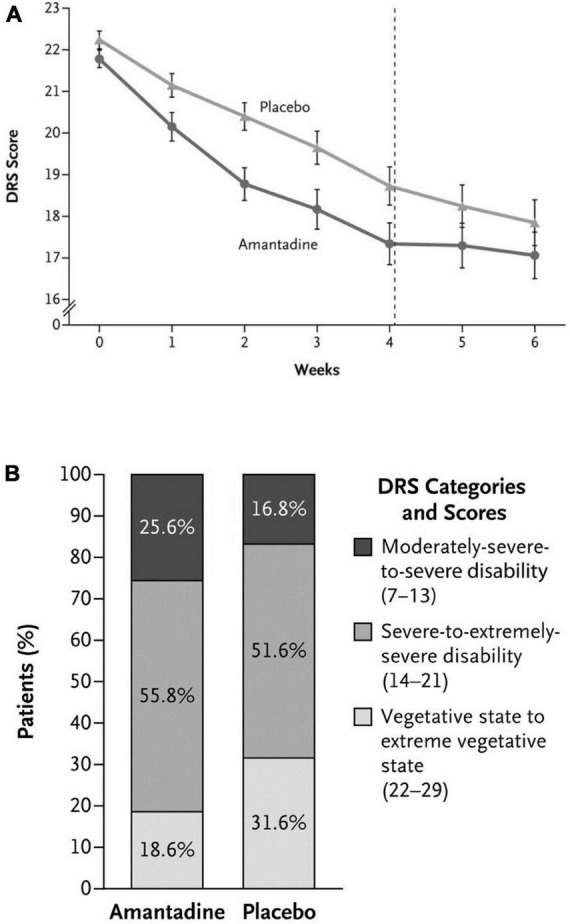
**(A)** Rate of functional recovery (DRS score) as a function of duration of treatment with amantadine compared to placebo in patients with severe TBI. DRS scores were improved significantly more rapidly following amantadine during the 4 week treatment period compared to placebo. On weeks 5 and 6 (washout interval), recovery rates in the amantadine group were significantly slower. Error bars indicate mean values ± SE. **(B)** Effects of amantadine treatment compared to placebo as a function of the category of functional disability (DRS score). After 4 weeks of treatment, the proportion of patients in a vegetative-to-extreme vegetative state was significantly lower in the amantadine group by post-hoc analysis.

Traumatic Brain Injury [TBI] and its associated neurological disabilities [decreased levels of consciousness, cognitive impairment] although not classically included as neurodegenerative diseases, share certain similarities with the latter that include favorable responses to amantadine treatment *via* well-established mechanisms of action. Furthermore, certain neurobehavioral sequelae of TBI such as hyperexcitability, disinhibition and agitation may also manifest improvements (Figure 4) that include metabolic activity in sagittal, coronal and axial planes following amantadine treatment (Giacino et al., 2018) accompanied by faster improvements in Disability Rating Score [DRS] values (Butterworth, 2020c). Improvements of executive cognitive ability concomitant with improved prefrontal cortical function determined by PET has also been reported in amantadine-treated MS patients (Kraus et al., 2005).

Adverse events reported during use of adamantanes for treatment of PD, AD, MS, TBI are relatively minor in severity and most commonly include confusion, light headedness, swelling of hands, legs, sleep disturbances [rarely].

## Conclusion

Close inspection of the published articles cited in this review reveals important links between neurodegenerative diseases and COVID-19 with respect to both basic pathophysiology and treatment with members of the adamantane family of agents. Both PD and AD are important age-related co-morbidities with the potential to impact on the severity of COVID-19 and, conversely, the symptoms of COVID-19 [fever, stress, fatigue] are known to aggravate gait abnormalities, tremor and effectiveness of L-Dopa in PD patients. Furthermore, enhanced antibody responses to coronaviruses have been demonstrated in CSF samples from patients with PD.

Links that are distinct from those encountered in PD are known to occur in AD where the presence of one of the two conditions may result in worsening of the other (Xia et al., 2021). Again, as for PD, severity of symptoms of both AD and COVID-19 are age-related and, interestingly, both are pathologically to mechanistic factors including the deposition of APP and to the activation of putative cell-death mechanisms that include excitotoxicity due to NMDA-receptor activated uptake of Ca^++^ in addition to microglial-mediated proinflammatory responses shared by COVID-19 and AD. The notion of a viral etiology in AD remains popular one version of which proposes that herpes simplex virus type 1 [HSV-1] rather than tau may cause the inter-neuronal trans-synaptic pathological cascade involved in the inter-cerebral progression of AD. It should also be noted that additional mechanisms have also been proposed; for example, some years ago, amantadine was found to improve resolution of the dysfunction of peripheral airways in influenza (Little et al., 1976) similar mechanisms could be implicated in the case of COVID-19.

Adamantanes [particularly amantadine and memantine] have evidence-based support from a series of randomized controlled trials [RCTs], some with associated meta-analysis for the treatment of PD and of L-Dopa-related dyskinesias in PD [amantadine] and for cognitive dysfunction in mild-to-moderate AD with US-FDA approval granted in both cases.

Accumulating evidence is now available in support of direct antiviral actions of adamantanes including [particularly amantadine and memantine] against coronaviruses including SARS-CoV-2. Results of initial clinical studies based upon limited patient numbers support the use of amantadine for the treatment of COVID-19 in patients even in the presence of pre-existing PD where benefit for both conditions was reported (Rejdak and Grieb, 2020). Similar reports indicate beneficial effects of memantine in COVID-19-infected patients with cognitive impairment (Rejdak and Grieb, 2020). Amantadine is also effective for the treatment of traumatic brain injury and its CNS complications and for the relief of fatigue in patients with MS (Giacino et al., 2018; Butterworth, 2020c).

The present review touches on other issues related for example, to the effects of prolonged exposure to SARS-CoV-2 on the efficacy and durability of amantadine and/or memantine commonly employed for the treatment of neurodegenerative disorders while bearing in mind that many of these disorders are themselves considered as co-morbidities for COVID-19. In this latter regard, given the recent mechanistic and therapeutic advances of significant antiviral action of amantadine, the possibility emerges whereby treatment of the neurological symptoms characteristic of the degenerative disorder as well as the co-morbidity associated with severe COVID-19 infection could be envisaged in a simultaneous manner. Indeed, the present review cites evidence from case reports and pilot studies that is consistent with such a possibility. It will be important to now confirm and extend these interesting findings in appropriate randomized clinical trials in the near future.

With regards to this latter eventuality, it is important to note that there are a number of clinical trials planned or currently ongoing registered in ClinicalTrials.gov relating directly to studies of the efficacy of amantadine for the treatment of COVID-19 summarized in Table 2.

## Author contributions

RB contributed to the design and the manuscript production, includes editing as following: Literature search of adamantanes for treatment of PD, AD, MS, and TBI, writing of the manuscript, reviewing of the manuscript, formatting of the manuscript, creation of figures, references list, compliance with author’s guidelines, and online submission.

## References

[B1] Ball M. J. Lukiw W. J. Kammerman E. M. Hill J. M. (2013). Intracerebral propagation of Alzheimer’s disease: strengthening evidence of a herpes simplex virus etiology. *Alzheimers Dement.* 9 169–175. 10.1016/j.jalz.2012.07.005 23159044 PMC3578985

[B2] Brison E. Jacomy H. Desforges M. Talbot P. J. (2014). Novel treatment with neuroprotective and antiviral properties against a neuroinvasive human respiratory virus. *J. Virol.* 88 1548–1563. 10.1128/JVI.02972-13 24227863 PMC3911624

[B3] Butterworth R. F. (2020a). Amantadine for the treatment of Parkinson’s disease and its associated dyskinesias. *J. Parkinsons Dis. Alzheimer Dis.* 7:7.

[B4] Butterworth R. F. (2020b). Amantadine, Parkinson’s disease and COVID-19. *Covid Perspect. Res. Rev.* 01 1–6.

[B5] Butterworth R. F. (2020c). Amantadine for the treatment of traumatic brain injury and its associated cognitive and neurobehavioural complications. *Pharmacol. Pharm. Res.* 3 1–5. 10.1089/neu.2018.5738 29969935

[B6] Butterworth R. F. (2021). Potential for the Repurposing of Adamantane Antivirals for COVID-19. *Drugs R D.* 21 267–272. 10.1007/s40268-021-00351-6 34152583 PMC8214976

[B7] Butterworth R. F. (2022). Memantine for the treatment of Alzheimer’s disease: novel mechanisms and future opportunities. *Neurol. Neurorehabil.* 4 17–20. 10.37532/22.4.2.17-20

[B8] Colombo B. Martinelli Boneschi F. Rossi P. Rovaris M. Maderna L. Filippi M. (2000). MRI and motor evoked potential findings in nondisabled multiple sclerosis patients with and without symptoms of fatigue. *J. Neurol.* 247 506–509. 10.1007/s004150070148 10993490

[B9] DeLuca J. Genova H. M. Hillary F. G. Wylie G. (2008). Neural correlates of cognitive fatigue in multiple sclerosis using functional MRI. *J. Neurol. Sci.* 270 28–39. 10.1016/j.jns.2008.01.018 18336838

[B10] Fishman P. S. Gass J. S. Swoveland P. T. Lavi E. Highkin M. K. Weiss S. R. (1985). Infection of the basal ganglia by a murine coronavirus. *Science* 229 877–879. 10.1126/science.2992088 2992088

[B11] Genova H. M. Rajagopalan V. Deluca J. Das A. Binder A. Arjunan A. (2013). Examination of cognitive fatigue in multiple sclerosis using functional magnetic resonance imaging and diffusion tensor imaging. *PLoS One* 8:e78811. 10.1371/journal.pone.0078811 24223850 PMC3815348

[B12] Giacino J. T. Katz D. I. Schiff N. D. Whyte J. Ashman E. J. Ashwal S. (2018). Practice guideline update recommendations summary: Disorders of consciousness: Report of the Guideline Development, Dissemination, and Implementation Subcommittee of the American Academy of Neurology; the American Congress of Rehabilitation Medicine; and the National Institute on Disability, Independent Living, and Rehabilitation Research. *Neurology* 91 450–460. 10.1212/WNL.0000000000005926 30089618 PMC6139814

[B13] Kishi T. Matsunaga S. Oya K. Nomura I. Ikuta T. Iwata N. (2017). Memantine for Alzheimer’s disease: an updated systematic review and meta-analysis. *J. Alzheimers Dis.* 60 401–425. 10.3233/JAD-170424 28922160

[B14] Kong M. Ba M. Ren C. Yu L. Dong S. Yu G. (2017). An updated meta-analysis of amantadine for treating dyskinesia in Parkinson’s disease. *Oncotarget* 8 57316–57326. 10.18632/oncotarget.17622 28915672 PMC5593643

[B15] Kraus M. F. Smith G. S. Butters M. Donnell A. J. Dixon E. Yilong C. (2005). Effects of the dopaminergic agent and NMDA receptor antagonist amantadine on cognitive function, cerebral glucose metabolism and D2 receptor availability in chronic traumatic brain injury: a study using positron emission tomography (PET). *Brain Inj.* 9 471–479. 10.1080/02699050400025059 16134735

[B16] Little J. W. Hall W. J. Gordon Douglas R. Hyde R. W. Speers D. M. (1976). Amantadine effect on peripheral airways abnormalities in influenza. *Ann. Int. Med.* 85 177–182. 10.7326/0003-4819-85-2-177 782310

[B17] Moriguchi T. Harii N. Goto J. Harada D. Sugawara H. Takamino J. (2020). A first case of meningitis/encephalitis associated with SARS-Coronavirus-2. *Int. J. Infect. Dis.* 94 55–58. 10.1016/j.ijid.2020.03.062 32251791 PMC7195378

[B18] Pilling K. Butterworth R. F. (2021). Amantadine for the treatment of fatigue in multiple sclerosis: systematic review and summary of the evidence base. *J. Mult. Sclerosis* 8:272.

[B19] Prasad S. Holla V. V. Neeraja K. Surisetti B. K. Kamble N. Yadav R. (2020). Parkinson’s disease and COVID-19: Perceptions and implications in patients and caregivers. *Mov. Disord.* 35 912–914. 10.1002/mds.28088 32304118 PMC7264599

[B20] Rejdak K. Grieb P. (2020). Adamantanes might be protective from COVID-19 in patients with neurological diseases: multiple sclerosis, parkinsonism and cognitive impairment. *Mult. Scler. Relat. Disord.* 42:102163. 10.1016/j.msard.2020.102163 32388458 PMC7190496

[B21] Roelcke U. Kappos L. Lechner-Scott J. Brunnschweiler H. Huber S. Ammann W. (1997). Reduced glucose metabolism in the frontal cortex and basal ganglia of multiple sclerosis patients with fatigue: a 18F-fluorodeoxyglucose positron emission tomography study. *Neurology* 48 1566–1571. 10.1212/wnl.48.6.1566 9191767

[B22] Schwab R. S. England A. C. Jr. Poskanzer D. C. Young R. R. (1969). Amantadine in the treatment of Parkinson’s disease. *JAMA* 208 1168–1170. 5818715

[B23] Singh Tomar P. P. Arkin I. T. (2020). SARS-CoV-2 E protein is a potential ion channel that can be inhibited by Gliclazide and Memantine. *Biochem. Biophys. Res. Commun.* 530 10–14. 10.1016/j.bbrc.2020.05.206 32828269 PMC7305885

[B24] Smieszek S. P. Przychodzen B. P. Polymeropoulos M. H. (2020). Amantadine disrupts lysosomal gene expression: A hypothesis for COVID19 treatment. *Int. J. Antimicrob. Agents* 55:106004. 10.1016/j.ijantimicag.2020.106004 32361028 PMC7191300

[B25] Takahashi M. Yamada T. Nakajima S. Nakajima K. Yamamoto T. Okada H. (1995). The substantia nigra is a major target for neurovirulent influenza A virus. *J. Exp. Med.* 181 2161–2169. 10.1084/jem.181.6.2161 7760004 PMC2192055

[B26] Toft-Bertelsen T. L. Jeppesen M. G. Tzortzini E. Xue K. Giller K. Becker S. (2021). Amantadine has potential for the treatment of COVID-19 because it inhibits known and novel ion channels encoded by SARS-CoV-2. *Commun. Biol.* 4:1347. 10.1038/s42003-021-02866-9 34853399 PMC8636635

[B27] Wu H. M. Tzeng N. S. Qian L. Wei S. J. Hu X. Chen S. H. (2009). Novel neuroprotective mechanisms of memantine: increase in neurotrophic factor release from astroglia and anti-inflammation by preventing microglial activation. *Neuropsychopharmacology* 34 2344–2357. 10.1038/npp.2009.64 19536110 PMC3655438

[B28] Xia X. Wang Y. Zheng J. (2021). COVID-19 and Alzheimer’s disease: how one crisis worsens the other. *Transl. Neurodegener.* 10:15. 10.1186/s40035-021-00237-2 33941272 PMC8090526

[B29] Zhou Y. Gammeltoft K. A. Galli A. Offersgaard A. Fahnøe U. Ramirez S. (2021). Efficacy of ion-channel inhibitors amantadine, memantine and rimantadine for the treatment of SARS-CoV-2 in vitro. *Viruses* 13:2082. 10.3390/v13102082 34696509 PMC8537953

